# Effects of Milk and Dairy on the Risk and Course of Inflammatory Bowel Disease versus Patients’ Dietary Beliefs and Practices: A Systematic Review

**DOI:** 10.3390/nu16152555

**Published:** 2024-08-03

**Authors:** Radoslaw Kempinski, Damian Arabasz, Katarzyna Neubauer

**Affiliations:** Department of Gastroenterology and Hepatology, Wroclaw Medical University, Borowska 213, 50-556 Wroclaw, Poland; radoslaw.kempinski@umw.edu.pl (R.K.); damian.arabasz@umw.edu.pl (D.A.)

**Keywords:** milk, dairy products, diet, inflammatory bowel disease, ulcerative colitis, Crohn’s disease

## Abstract

Background: The role of the environment in the pathogenesis of inflammatory bowel disease (IBD) is undisputed, especially in light of numerous epidemiological data showing the increasing prevalence of IBD worldwide. Although no specific environmental factors have been identified, the diet has received the most attention as a potential modifier of the onset and course of IBD and as a therapeutic intervention. The Westernization of the diet is repeatedly cited as a crucial aspect of the change in IBD prevalence, but data on the impact of diet on the course of IBD are still limited and the effectiveness of dietary interventions remains uncertain. Milk remains one of the most discussed dietary agents in IBD. Materials and methods: We performed a systematic review of the literature published between January 2010 and March 2024 on three databases, Pubmed, Web of Knowledge, and Embase, to assess the impact of milk and dairy products on the risk and course of IBD, as well as patients’ dietary beliefs and practices. Results: We included 37 original studies in our review. Conclusions: There is no clear evidence that milk and dairy products influence the incidence and course of IBD. The studies that assess this issue are characterized by great heterogeneity. Milk and dairy are among the most commonly excluded foods by patients with IBD, which may have clinical implications.

## 1. Introduction

Inflammatory bowel disease (IBD) is an umbrella term for the chronic, unpredictable, and potentially devastating conditions of the gastrointestinal tract encompassing ulcerative colitis (UC) and Crohn’s disease (CD). The pathogenesis of IBD is complex and implicates the interactions between the microbiome and immune system in genetically predisposed individuals in the presence of still poorly defined environmental exposures [1,2,3]. The significance of environment seems to be undisputable, especially in the light of numerous epidemiological data indicating the increasing prevalence of IBD worldwide leading to the recognition of IBD as an emerging global disease [4]. Debated environmental factors include dietary agents as the number of people with IBD across the world’s regions adopting the Western diet is rising [5,6,7]. Additionally, studies on migration show differences in the incidence, prevalence, and disease phenotype between migrants and indigenous populations [8]. A recently published large study from India, a region considered to have a low IBD incidence, also identified environmental factors as a risk factor for IBD [9]. The Western diet is considered to be pro-inflammatory [10], and it negatively affects the gut microbiota and the immune system. One of its key features is an overall higher energy intake, a high consumption of processed food, meat, refined grains, sugar, salt, alcohol, high-fat dairy products, and a low consumption of fruits and vegetables [11]. However, data on the role of specific dietary components in the pathogenesis of IBD remain limited [12,13] and carrying out studies is challenging because of the complex nature of the relationships between diet, genetics, and the microbiome [14]. Still, diet remains the focus of researchers’ attention as it may impact not only the development of IBD but also its course, and it is implemented in the therapy. In addition, the effectiveness of dietary interventions in IBD remains uncertain. For instance, a review of 18 RCTs with 1878 participants was not able to provide evidence supporting the role of the dietary approaches examined in IBD [15].

Milk and dairy are significant elements of a healthy and balanced diet as sources of nutrients and energy. According to a WHO report, cow’s milk consumption is estimated to be 10–212 kg per person per year [16]. Despite the controversies regarding the intake of the milk of other species by adults, there is no clear data on its harmful effects. Additionally, a growing body of evidence indicates that dairy food consumption does not increase the concentration of the inflammatory biomarkers [17,18,19]. On the contrary, multiple studies have documented a significant anti-inflammatory effect of dairy [20]. Furthermore, dairy consumption in the frame of a healthy dietary pattern can be protective against the occurrence of diabetes mellitus or cardiovascular diseases [21]. In summary, milk has numerous activities, including anti-carcinogenic, anti-inflammatory, anti-oxidative, anti-adipogenic, anti-hypertensive, anti-hyperglycemia, and anti-osteoporosis [22].

The impact of milk and dairy consumption on the human microbiome is multifactorial. The human microbiome is acquired at birth, undergoes dynamic changes during childhood, and remains a relatively stable structure in adulthood. The composition of the microbiome varies but it appears that the Bacteroides/Fimicutes ratio may determine the occurrence of diseases such as obesity or IBD [23]. The active components of milk including oligosaccharides and whey proteins, especially lactoferrin, lysozyme, and alpha-lactalbumin can favorably influence components of the microbiome. Milk oligosaccharides show prebiotic effects, and promote the proliferation of Bifidobacterium and Lactobacillus strains [24,25]. Lactoferrin increases the amounts of Bifidobacterium and Lactobacillus in the intestine and reduces the abundance of Escherichia coli in the colon, which improves the integrity of the intestinal barrier and enhances the intestinal immune system. Alpha-lactalbumin has been shown to promote the growth of short-chain fatty acid-producing bacteria in the intestine and increase the favorable Bacteroides/Fimicutes ratio [26,27]. Lysozyme is a component of colostrum that affects the composition of the intestinal microbiome: it reduces the abundance of Escherichia coli in the intestine, which can reduce inflammation in the intestine and its damage. It has a beneficial effect on the Bacteroides/Fimicutes ratio in the gut [28,29].

Yet, milk elimination is justified in the presence of adverse food reactions. They are common in children and can occur in 6–28% of them. Adverse food reactions are divided into immune-mediated food allergies and non-immune-mediated food intolerances. The prevalence of food allergies ranges from 1 to 10% and cow milk is the most common cause of food allergy in young children [30].

In turn, milk-related food intolerances result from a decreased activity of lactase, an enzyme in the epithelium of the small intestine, and may be primary, regulated by genetic mutations, or secondary, due to the damage of the epithelium.

Lactose intolerance is widespread worldwide, affecting about two-thirds of the population. The prevalence varies by geographic region, being lowest in western and northern Europe and highest in the Middle East. A meta-analysis showed that the prevalence of lactose intolerance is more common in patients with inflammatory bowel disease, particularly CD involving the small intestine. This may be related to the degree of mucosal damage, motility disorders, and bacterial overgrowth. In other cases, the ethnic and geographic considerations mentioned above are responsible for lactose intolerance [31,32].

Undigested lactose reaching the large bowel causes diarrhea and/or bloating and, therefore, may cause the symptoms in IBD patients with lactase deficiencies to worsen. However, patients may tolerate dairy products with less lactose, e.g., yogurt, or without any lactose, e.g., hard cheeses [33].

It also has to be noticed that the nutritional quality of most plant milk imitating drinks is lower than cow’s milk in terms of the levels of protein, vitamins, and essential minerals. The consumption of non-fortified plant drinks may be related to the risk of deficiencies of calcium, zinc, iodine, vitamins B2, B12, D, A, and indispensable amino acids [34].

Given the prevalence of lactose intolerance, alternative dietary strategies targeting this patient group, together with improved food labeling, are of great interest. It is worth noting that, in addition to lactose-free and plant-based products obtained, for instance, from rice, soy, oats, or nuts, the use of prebiotics is also proposed. The exclusion of milk and dairy products from the diet is associated, among other things, with the risk of calcium deficiency and the development of osteopenia, and therefore nutritional education plays a key role [35,36].

Milk and dairy products contain bone-beneficial nutrients, such as protein, calcium, and phosphorus, but also D-galactose, which was associated with premature aging in experimental studies. Furthermore, data on milk intake and the risk of osteoporosis and hip fracture are inconsistent. For instance, in a recent systematic review and meta-analysis, a greater intake of milk and dairy products in cohort studies was not associated with a lower risk of osteoporosis and hip fracture [37]. In turn, a reduced risk of fracture with higher milk consumption was observed in the USA, but not in Scandinavia [38]. Randomized controlled trials have demonstrated that the presence of milk in the diet may potentially prevent bone loss through the complex interplay between the calcium–vitamin D–parathormone axis, and growth hormone/insulin-like growth factor-1 axis [39]. Mammalian milk is an abundant source of lactoferrin, an iron-binding glycoprotein. Lactoferrin possesses multidirectional biological roles including antibacterial and anti-inflammatory activity, intestinal barrier preservation, immune modulation, and maintenance of intestine homeostasis [40]. However, dietary lactoferrin is enzymatically degraded in the gastrointestinal tract, and as a consequence, loses its functional properties [41].

Guidelines on the standard diet for IBD are also inconsistent and scanty. For instance, ESPEN recommends a standard diet for patients with IBD in remission, highlighting the lack of strong evidence supporting the elimination of dairy foods, and regular screening for malnutrition [42]. Similarly, the British Dietetic Association discourages self-directed non-evidence-based exclusion diets as they can promote nutritional deficiencies and a low diet quality [43]. In parallel, there are multiple efforts towards the creation of dietary approach, oriented toward reducing exposure to dietary components that have adverse effects on the microbiome and intestinal barrier, which may induce remission in patients with IBD [44]. One example is the CD treatment-with-eating diet (CD-TREAT), a nutritional therapeutic program replicating the exclusive enteral nutrition (EEN) with an ordinary food diet, which is known as an effective therapy for CD flare in children [45]. Furthermore, the CD exclusion diet (CDED), a whole-food diet coupled with partial enteral nutrition (PEN) was effective in inducing remission in children with CD [46]. In the pilot RCT, CDED with or without PEN was effective for the induction and maintenance of remission in adults with mild-to-moderate biologically naive CD. It is worth noting that, in the maintenance phase of CDED, one yogurt per day is allowed and other dairies are allowed on weekends [47].

Hence, we conducted a systematic review of the recent literature to assess the relationship between the consumption of milk and dairy products and IBD risk, the impact of consumption of milk and dairy products on IBD course, and patients’ beliefs and practices concerning milk and dairy products.

## 2. Material and Methods

**Figure 1:**
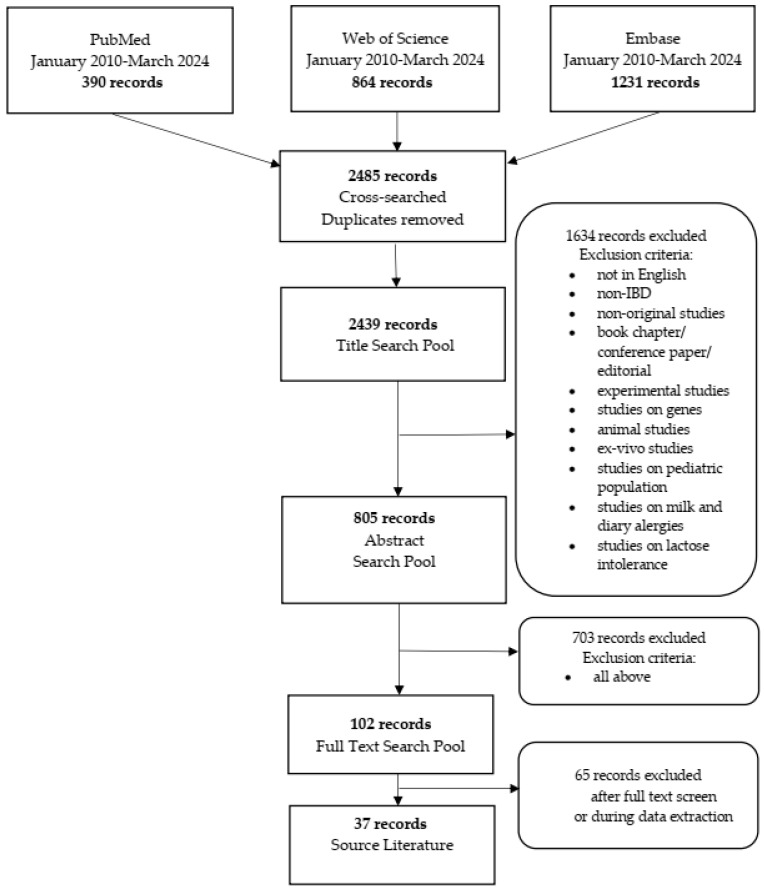
The selection process according to PRISMA guidelines.

We searched three publication databases: PubMed, Web of Knowledge, and Embase. Combinations of the following keywords (“dairy product*” OR “milk” OR “yogurt” OR “cheese” OR “diet*” AND (“Crohn’s disease” OR “ulcerative colitis” OR “inflammatory bowel disease*” OR “IBD”) were applied. The search was limited to publications published between January 2010 and March 2024. The asterisks allowed us to retrieve records where query words appeared with suffixes (e.g., disease|s). The reporting of this systematic review was guided by the standards of the Preferred Reporting Items for Systematic Review and Meta-Analysis (PRISMA) Statement [48]. Duplicate records from the databases were removed before the first eligibility screening. Exclusion criteria were as follows: experimental studies (including animal studies and in vitro research), studies in children, studies on milk and dairy allergies; studies on lactose intolerance; studies on genes; non-IBD, non-cow milk; non-original articles, and non-English language, abstracts, and posters. The selection process is shown Figure 1. The Evidence-Based Medicine (EBM) pyramid was used to assess the quality of the study [49].

## 3. Results

We identified 37 original studies fulfilling the inclusion criteria:

1. Eight studies (*n* = 8) on the risk of milk and dairy consumption on the occurrence of IBD;
2. Twenty-seven studies (*n* = 27) on the dietary beliefs and practices regarding milk and dairy products (one study covered beliefs and practices regarding milk and dairy consumption and the impact of milk and dairy consumption on the IBD course);
3. Three studies (*n* = 3) on the impact of milk and dairy consumption on the IBD course.

### 3.1. Interpretative Synthesis of Data: Milk and Dairy Products and the Occurrence of Inflammatory Bowel Disease

We identified eight studies that assessed the impact of diet, including the consumption of milk and dairy products, on the occurrence of IBD (Table 1). Two studies have a special significance, as they were prospective, multi-center, population-based, and performed in large cohorts. In the European Prospective Investigation into Cancer and Nutrition (EPIC) study, which was conducted in 12 centers and limited to Europe, the possible protective influence of milk consumption on the occurrence of both CD and UC was reported [50]. Yet, the recently published results of the Prospective Urban Rural Epidemiology (PURE) study, carried out in centers around the world, did not reveal an association between milk consumption and IBD [51].

The study by Opstelten et al. [50] was conducted in the EPIC cohort. The EPIC study aimed to assess the relationship between diet and the incidence of cancer and chronic diseases. The authors evaluated dietary intake by using validated food frequency questionnaires (FFQs) composed of approximately 200 food items and nine frequency categories of intake. Dairy included milk (whole fat, skimmed, semi-skimmed, and unspecified), yogurt (natural and flavored products and fermented milk in Denmark and Sweden), and cheese (fresh, fermented, matured cheese products). During the observation, 110 individuals (72.7% female) developed CD at a mean age of 55.4 years, and 244 participants (57.4% female) developed UC at a mean age of 57.5 years. The median time between enrolment and diagnosis was 5.1 years (range 1.5–14.3 years) and 4.8 years (range 1.5–15.7 yr) for CD and UC, respectively. No significant relationships according to quartiles or trends between the intake of total or individual dairy products and the development of either CD or UC were found. Still, compared with non consumers, participants consuming milk had significantly decreased odds of developing CD but did not have significantly reduced odds of developing UC.

Using the PURE cohort, the authors studied the association between ultra-processed food intake and the risk of developing IBD. Standardized questionnaires were applied and usual food intake was assessed using country-specific FFQs. Among the studied food items was dairy. Dairy intake was categorized into <1 serving/day, 1 to <2 servings/day, or ≥2 servings/day. Participants were enrolled in 21 countries and there were 467 cases of IBD diagnosed during a median follow-up of 9.7 years: 192 cases in Europe and North America, 33 cases in South America, 25 in Africa, 103 in the Middle East, 94 in South Asia, 1 in Southeast Asia, and 19 in China. Intake of dairy, but also white meat, unprocessed red meat, starch, fruit, vegetables, and legumes, was not associated with the risk of IBD. However, a higher consumption of ultra-processed food was associated with a higher risk of IBD. The study had strength in the form of its multinational and prospective nature and also a large cohort. Individuals who were diagnosed with IBD within one year of the baseline questionnaire were excluded. Among the limitations, age of the participants (35–70) may be listed as IBD tends to develop in younger age groups [51].

In turn, van der Sloot et al. [52], in a case–control study on 647 IBD patients among the novel environmental factors related to IBD, identified self-reported cow’s milk hypersensitivity. However, it was related only to CD. Han et al. [53] in a secondary analysis of the National Health Interview Survey 2015, found out that consumption of cheese and ice cream was associated with IBD. Yet, drinking milk was related to a lower likelihood of having IBD. In turn, Li et al. suggested that consumption of whole-milk fat may protect against CD, compared with skimmed milk [54].

The few studies identified have significant limitations (small study group, retrospective nature of the work, study design preventing conclusions) and hence do not provide answers to the questions we asked in our review [55,56,57].

In summary, the association between the risk of IBD and milk and dairy product consumption remains unclear. The results of two prospective studies conducted in large cohorts are inconsistent. One study did not demonstrate an association between milk/dairy consumption and IBD risk [51], while the other study demonstrated a tendency for a protective effect of milk and dairy [50]. Further studies are warranted.

**Table 1:** Impact of milk and dairy products consumption on the risk of IBD.

Author	Study Country Quality Level *	Methods	Patients	Findings
Maconi et al. [55], 2010	Case–control Italy D	Questionnaire previously used for studies on the relationship between cancer and diet; consumption of foods and beverages was subdivided into tertiles (low, moderate, and high consumptions)	41 UC, 42 CD, 160 sex- and age-matched healthy blood donors; patients not changing their dietary habits (26 UC and 25 CD) were analyzed concerning the IBD risk	CD was significantly associated with high consumption of cheese (OR = 3.7, 95% CI: 1.14–12.01); an increased risk of ileal or ileocolonic CD was associated with high cheese consumption (OR = 2.61, 95% CI: 1.26–5.41). Remark: to conclude abiut the effect of milk and dairy consumption on the risk of IBD is impossible.
Opstelten et al. [50], 2016	Prospective cohort study; 12 centers in Denmark, France, Germany, Greece, Italy, the Netherlands, the United Kingdom, and Sweden C	FFQ for the intake of total and specific dairy	110 CD and 244 UC patients from 401,326 participants of the EPIC cohort	Compared with the lowest quartile, the ORs for the highest quartile of total dairy products were 0.61 (95% CI, 0.32–1.19) for CD and 0.80 (95% CI, 0.50–1.30) for UC. Compared with non-consumers, participants consuming milk had significantly reduced odds of CD (OR 0.30, 95% CI, 0.13–0.65) and non-significantly reduced odds of UC (OR 0.85, 95% CI, 0.49–1.47).
Han et al. [53], 2020	A secondary analysis of National Health Interview Survey 2015 United States C	Survey	In the total population of 103,789 individuals 33,672 adults (44.76% males; 55.24% females) 454 responders (1.35%) were ever told by health professionals that they had IBD. The prevalence of IBD among estimated US adults is 1.28% (95% CI 1.27–1.28)	Drinking milk was associated with a smaller likelihood of IBD (OR 0.70, 95% CI 0.497–0.998). A significant association between IBD and consumption of cheese (OR = 1.006, 95% CI [1.0021–1.0104], *p* = 0.003), ice cream (OR = 1.011, 95% CI [1.0022–1.0203], *p* = 0.015), was shown.
Van der Sloot et al. [52], 2020	Case–control study The Netherlands D	The validated GIEQ identifying factors through different stages of life using 844 items	674 IBD patients of the 1000IBD cohort, frequency-matched based on sex and age with 1348 controls from the population-based Lifelines Cohort Study Mean age at study inclusion 50.4 years	Cow’s milk hypersensitivity (OR 5.87; 95% CI 2.72–12.68) was related to CD.
Preda et al. [57], 2020	An observational, retrospective, case–control study Romania, Belgium D		76 Romanian and 53 Belgian patients	Lower intake of yogurt by IBD patients (25.6 vs. 43.8%) Remark: small, retrospective study; to conclude the effect of milk and dairy consumption on the risk of IBD is impossible.
Bikbavova et al. [56], 2021	Retrospective case–control study Russia (Western Siberia) D	World Health Organization countrywide integrated noncommunicable diseases intervention questionnaire	81 UC (42 men and 39 women, age 18–79 years); 39 controls (healthy volunteers, 14 men, 25 women, aged 22–81 years)	UC patients poorly tolerated milk and fermented milk products before the first disease symptoms more frequent compared to the control group (27.2 ± 4.9% vs. 7.7 ± 4.3%, *p* = 0.011) Remark: small, retrospective study; to conclude about the effect of milk and dairy consumption on the risk of IBD is impossible
Narula et al. [51], 2021	PURE study 21 countries ** C	Country-specific validated FFQs	The PURE study cohort of 116,087 adults aged 35–70 years. 90 CD and 377 UC 273 women The mean age of IBD patients was 50.5 years.	Intake of dairy was not associated with incident IBD.
Li et al. [54] 2021	United Kingdom C	A two-sample Mendelian randomization analysis	20,200 whole-milk consumers and 67,847 skimmed-milk consumers from the UK Biobank	The genome-wide association study identified five lead nucleotide polymorphisms associated with whole-milk preference. Whole-milk preference significantly decreased the risk of IBD (b = 1.735, *p* = 0.048) and CD (b = 2.549, *p* = 0.032), but had no significant effect on the risk of UC (b = 1.002, *p* = 0.44).

* C: cohort study; D: case–controlled study; EPIC, European Prospective Investigation into Cancer and Nutrition; PURE, Prospective Urban Rural Epidemiology; FFQs, food frequency questionnaires; IBD, inflammatory bowel disease; CD, Crohn’s disease; UC, ulcerative colitis; US, United States of America; GIEQ, Groningen IBD Environmental Questionnaire; ** Argentina, Bangladesh, Brazil, Canada, Chile, China, Colombia, India, Iran, Malaysia, Palestine, Pakistan, Philippines, Poland, South Africa, Saudi Arabia, Sweden, Tanzania, Turkey, United Arab Emirates, Zimbabwe.

### 3.2. Interpretative Synthesis of Data: Dietary Beliefs and Practices Related to Milk and Dairy Consumption of Patients with Inflammatory Bowel Disease: The Impact of Milk and Dairy Consumption on the IBD Course

The available studies vary in several respects, including number of patients and their characteristics, geographical location, methods used to assess milk and dairy intake, and the overarching purpose for which they were conducted, making them difficult to synthesize. Nine of the twenty-seven studies (n = 9) were conducted in groups of less than 100 patients. Three studies were conducted in Asian countries (Korea [58], India [59], Taiwan [60]), one in Brazil [61], and one in a British Asian population [62]. Thus, these studies included ethnic groups with a higher prevalence of lactose intolerance, which may influence patients’ beliefs and practices related to milk and dairy consumption. Nevertheless, studies were consistent with the widespread belief among IBD patients that milk and dairy products have a detrimental effect on their disease course. The dietary habits of IBD patients were consistent with patients’ beliefs and milk and dairy are among the most frequently eliminated or restricted dietary items. The characteristics of the studies are shown in Table 2.

In one of the biggest studies, dietary patterns differed between subgroups (CD, UC, UC-pouch, and CD-ostomy patients), but self-reported perceptions of what was worsening and what was improving symptoms did not [63]. One of the food items that improved the symptoms reported by all subgroups of patients was yogurt. At the same time, most subgroups reported milk and dairy as a food worsening the symptoms. Furthermore, among the other food groups, patients ate less or more milk, cheese, and ice cream when they reported that these items worsened or improved their symptoms. Additionally, the authors noticed some differences in the dietary habits between the IBD subgroups. For instance, CD patients with an ostomy consumed more milk than patients without, and CD patients with active disease reported consuming significantly less milk than patients in remission. The strengths of the study include a large cohort of motivated patients and applying a questionnaire that was already used in the larger studies. Among the limitations, lack of phenotype classification and self-reported disease activity evaluation are listed. In another study Peters et al. [64], in a large cohort of almost 500 IBD patients and almost 1300 control individuals, reported that both UC and CD participants consumed less dairy than healthy controls. This observation was made in patients with active and non-active diseases. UC patients ate more dairy than CD patients. Moreover, almost 40% of the patients in remission and almost 90% of the patients with active disease had a protein intake below recommendations.

There are also many studies conducted in smaller patient samples demonstrating similar findings. For instance, Guida et al. [65] reported that milk and dairy were listed as foods perceived as symptom triggers by patients with IBD by over one-third of patients, after spicy food (49.1%) and seasoned food (38.3%). Patients avoided certain foods in active and not-active disease. From among specific diets followed by 33.6% of patients, the most frequently adopted was lactose-free. Still, nearly 17% of individuals had performed a diagnostic evaluation to confirm or exclude the presence of lactose intolerance or a milk protein allergy. The reason for the dairy restriction given by almost 70% of patients was their suspected role in triggering symptoms. In turn, a lactose-free diet was recommended by the treating physicians only for 14.3% of patients and was more common in patients with CD. The study was performed in a cohort of patients with IBD, with homogeneous ethnic origins and food cultures. Disease activity was assessed with questions about disease activity perceived by the patient as well as with the partial Mayo Score for UC and Harvey Bradshaw Index for CD. However, among the limitations, the authors identified discrepancies between disease activity assessed with clinical scores and patients’ perception as well as recall bias. In turn, Lopes et al. [61] observed that 52.3% of the patients reported alterations in the consumption of dairy after IBD diagnosis. First of all, almost 65% of the patients described dairy restrictions. Expectedly, the most common background was the exacerbation or onset of symptoms (45.5%); however, almost 40% of patients followed the recommendations of health professionals. They found that 52% of IBD patients had a low dairy product intake, and 91% had inadequate dietary calcium intake. The limitations of the study consisted of the size of the sample and not considering comorbidities that could influence dietary habits. As expected, Crooks et al. [66] found that milk was among the most frequently reported dietary triggers for disease relapse and one of the most commonly avoided dietary products in a group of 255 British South Asians with IBD.

We also assessed four studies conducted in Poland for the review. Krela-Kazimierczak et al. [67] carried out the research in a group of 208 IBD patients focused on bone mineral density (BMD) and the occurrence of osteopenia and osteoporosis (OP), as well as their correlation with the consumption of dairy. The study showed that almost 90% of IBD patients consumed milk before a diagnosis of the disease, and the intake significantly decreased afterward, which, in the opinion of authors, may further impair BMD. Among the limitations of the study, the authors listed the small size of the studied group and the lack of a quantitative determination of the milk and dairy products consumed. Sienkiewicz et al. [68] reported that patients with IBD consumed milk and dairy products less frequently than the control group. Furthermore, a statistically significant positive association was found between milk consumption and the occurrence of diarrhea in IBD patients. The authors found a higher mean reported frequency of milk consumption in the group with diarrhea versus the group without diarrhea (0.83 ± 1.10 vs. 0.45 ± 1.03). Almost 40% of patients avoided milk and milk was among ten the most frequently avoided foods. The limitations of the study are related to the lack of FFQ validation, the cross-sectional study design, demographic differences between both groups, and the seasonal character of diet. In another study, ref. [69], despite there being no difference between milk and dairy intake between participants with UC and healthy volunteers, the authors noticed that 32% of UC males and 20% of control males reported the exclusion of dairy products. In another study from Poland [70] almost 50% of patients believed that milk and dairy may impact the disease course and in turn, the vast majority were on a lactose-free diet.

Lim et al. [58] demonstrated, that milk and dairy products were restricted by almost 33% of the IBD patients, being the most commonly restricted food. Furthermore, malnutrition was more frequent in the food-exclusion group compared to the food-non-exclusion group (*p* = 0.007). Similarly, calcium intake was lower in the food exclusion group (*p* = 0.002).

In turn Vidarsdottir et al. [71], in a small sample of IBD patients, also found that milk and dairy were connected to the aggravation of the symptoms and therefore the commonly excluded food items. Walton et al. [72] reported that 13% of UC patients excluded milk and dairy products, whereas 25% avoided them. Interestingly, most of the participants avoided these diet components during both flare-ups and remission (57%), whereas only 26% avoided them during flare-ups.

In the Canadian cohort study, Vagianos et al. [56] reported that 12% of patients constantly avoided milk and dairy products. Food avoidance was more common in patients with active disease. Additionally, the authors noticed, that the diet of patients with IBD differed as compared to the non-IBD population; however, deficiencies were present in both groups. Further, IBD patients consumed more milk than the control group, even though 15% restricted milk due to health professionals’ advice.

Casanova et al. [73] carried out a study in a big cohort of IBD patients in Spain. The majority of patients (77%) declared using food restrictions to prevent disease relapse; for instance, 23% of patients avoided dairy products. Disease activity influenced the dietary restrictions and even more patients (86%) avoided some foods when they had the flare. They found that 36% of patients avoided dairy products during a flare. Additional findings from the study regarding patients’ beliefs are also significant. Almost 70% of the patients believed that diet can induce a disease flare and almost 90% believed that they would benefit from professional advice.

Larusa et al. [74] carried out a study on self-prescribed dietary restrictions in 90 IBD patients. The majority of patients (70%) reported dietary avoidance, and among them, 84% avoided dairy products. Moreover, avoiding dairy products was a significant risk factor for low BMD, and dietary restrictions were also associated with a lower BMI.

Opstelten et al. [75] carried out a study in patients with longstanding IBD by using an FFQ. It has to be highlighted that disease activity was assessed with endoscopy or medical records at the time when the questionnaire was completed. The control group was recruited from participants in the population study Nutrition Questionnaires plus. The authors reported that IBD patients had a lower normal consumption of dairy products as compared to controls. What makes the study exceptional is that the association between macronutrient intake and IBD relapse was evaluated. Of the study participants, 82% of were in clinical or endoscopic remission at baseline, and with a median follow-up of 29 months, 30% of them developed a relapse. In multivariable logistic regression analyses, an inverse association of developing a relapse was found with a saturated fatty acid, total fat, and monounsaturated fatty acid intake while a positive association was found for dietary fiber intake. Among the limitations of the study authors listed the following: the IBD patients were recruited from the group undergoing endoscopic surveillance for colorectal cancer; therefore, they had longstanding disease with colon involvement; participants of the control group, were less frequently smokers and received a relatively higher level of education; the FFQs covered intake during the preceding month only. The undebatable strengths of the research are, firstly, the endoscopic evaluation of the disease activity, and secondly, studying the relationship between dietary intake and disease course.

Bach et al. [76], in a small sample of UC patients, observed that 60% restricted their consumption of dairy products. Yet, besides the small number of patients, the study had had other limitations; for instance, a broad range of disease duration (1–35 years).

Zallot et al. [77] assessed not only dietary beliefs and practices in adult IBD patients but also their impact on social life. 15.6% of patients believed that diet could initiate the disease, whereas 57.8%, that food can cause a relapse. 25.7% of patients thought, that restricting dairy product consumption may prevent disease relapse. Additionally, patients excluding dairy products also excluded spicy food, carbonated beverages, fat, vegetables, and fruits in 86.6%, 62.7%, 59.7%, 58.2%, and 56.7% of cases, respectively. Consequently, the study demonstrated that dietary exclusions had an impact on social life.

Triggs et al. [78] in a large cohort study of CD patients in New Zealand showed that among the top ten foods for which the most participants reported beneficial effects was yogurt. However, 18.1% reported adverse effects and 62.8% no difference after yogurt consumption. Whereas milk and dairy products were not located among the top ten foods reported to have a detrimental effect, in over 40% of subjects, cream was causing symptom enhancement, and full-fat milk as well as cheese were tolerated by significantly fewer subjects than low-fat milk.

In the study by Vagianos et al. [79] over 60% of study participants believed that food may trigger flares, and 25% of those with active disease believed that a dietary compound may have triggered their current flare.

Marsh et al. observed that almost all IBD patients reported food avoidance, with more foods avoided during the flare. Lactose was avoided by forty percent of patients in active disease and one-third in remission. Gastrointestinal symptoms were the most common explanations for dietary restrictions [80].

In summary, IBD patients are convinced that milk and dairy negatively affect their gastrointestinal symptoms. Almost 50% of patients (45.8–48%) reported a relationship between the first IBD symptoms and milk and dairy consumption, whereas 34–87% reported a connection between those foods and IBD flare. It was found that 12–84% of IBD patients introduced the restriction or elimination of milk and dairy consumption. Almost 70% of patients had symptom relief after these dietary restrictions. Interestingly, in two large studies, the beneficial impact of yogurt consumption on IBD symptoms was reported [63,78].

**Table 2:** Dietary beliefs and practices related to milk and dairy consumption in patients with inflammatory bowel disease.

Author Year	Study Design Country Quality Level *	Methods (a Tool Used to Assess Milk and Dairy Consumption)	Patients	Beliefs	Practice
Vagianos et al. [81] 2006–2007	Cohort study Canada C	Surveys semi-annually and annual in-person interviews	319 IBD patients from the University of Manitoba IBD Cohort Study Diets of those with IBD (n = 256) were compared with a matched, non-IBD Canadian cohort	Potential “gastrointestinal upset” was the reason to avoid milk and milk products in 51% of patients.	12% always avoid milk and milk products. 29% normally eat milk/milk products but avoid them when the disease is active.
Triggs C. et al. [78] 2010	Case–controlled New Zealand D	A dietary questionnaire (257 food items); self-reported dietary tolerances and intolerances.	446 CD	Yogurt beneficial:19.1%; adverse 18.1%; Dairy products beneficial: 5.6%; adverse: 20.5%; no difference 46.7%	
Cohen A. et al. [63] 2013	Internet-based cohort study United States C	A semi-quantitative FFQ developed by National Cancer Institute quantified the average daily consumption in the prior month of several food items	2329 IBD: 1121 CD ^a^ 597 UC 405 CD-O 206 UC-P approximately 70% females median age 42–49 years 58–73% of participants reported having minimal disease activity	yogurt improved symptoms: CD 108 vs. 7 ** UC 54 vs. 3 ** CD-O 26 vs. 0 ** UC-P 19 vs. 0 **; milk worsened symptoms: CD 105 ** vs. 6 UC 49 ** vs. 0 CD-O 28 ** vs. 5 UC-P 14 # vs. 2 dairy worsened symptoms: CD 94 ** vs. 3 UC 56 ** vs. 1 CD-O NR UC-P 12 # vs. 0	Patients ate less or more milk, cheese, and ice cream when they reported that these items worsened or improved their symptoms (*p* < 0.05). CD patients with disease flare consumed less milk than those in remission (OR 0.78, 95% CI 0.62–0.97).
Zallot C. et al. [77] 2013	Cross-sectional France E	A questionnaire by the Departments of Gastroenterology and Diabetes and Nutrition of the Nancy University Hospital	244 IBD 60.7% females 72.5% CD	27.5% perceived milk to be a risk factor for relapse; 27.4% believed a dairy-free diet can improve symptoms during relapse	Patients excluding dairy products also excluded spicy food, carbonated beverages, fat, vegetables, and fruits in 86.6%, 62.7%, 59.7%, 58.2%, and 56.7% of cases, respectively.
Limdi et al. [82] 2013–2014	Single-center, prospective study UK D	Prospective questionnaire	205 UC 156 CD	48% of IBD patients reported the link between diet and disease onset, while 57% between diet and disease flare Worse milk and milk products worsened symptoms in 16% of patients.	
Bach et al. [76] 2014	Denmark D	Semi-structured qualitative interview	25 UC 46% males 46.7 ± 15.6 years		60% of the patients did not drink milk at all; restrictions based on personal experience.
Lopes et al. [61] 2014	Cross-sectional study Brazil E	Quantitative FFQ	44 UC 21 CD 61.5% females Mean age 44.8 ± 13.5 years	45.5% of patients reported a relation between exacerbation or onset of symptoms and cow’s milk and dairy consumption.	64.7% of patients restricted milk and dairy products consumption. There was no difference between UC and CD patients.
Vidarsdottir et al. [71] 2016	Cross-sectional Iceland E	3-day food record	43 CD 35 UC 35 men and 43 women aged 18–74 years	87% of participants reported that diet influences gastrointestinal symptoms and 72% had changed their diet accordingly	60% restricted dairy products due to negative effects on symptoms.
Casanova et al. [73] 2017	Multicentre, observational, prospective study conducted at 30 centers Spain E	A questionnaire by the IBD Unit and the Endocrinology and Nutrition Department	1271 patients 60% CD 51% females median age of 45 years		23% of patients avoided dairy to prevent relapse of the disease, 36% avoided dairy during relapse.
Lim et al. [58] 2018	Cross-sectional Korea E	Survey	61 CD 43 UC 2 groups: exclusion diet (n = 49) and non-exclusion diet (n = 55)		In the exclusion diet group, milk and dairy were the most frequently restricted foods.
Larussa et al. [74] 2019	Single-center Italy E	Self-prescribed dietary restrictions in patients with IBD	67 UC 23 CD		27% of patients avoided milk alone. 84% of patients avoided at least one dairy product.
Opstelten J. et al. [75] 2019	Cohort study The Netherlands C	Self-completed semiquantitative FFQ	165 patients with longstanding IBD vs. 1469 Dutch population-based study		In IBD patients consumption of dairy products (except cheese) was −36.3 g/d lower than in control group.
Głabska et al. [69] 2019	Matched case–control study Poland D	Three-day dietary record	44 male UC patients in remission		Consumption of milk and dairy beverages did not differ between UC patients and controls.
Marsh et al. [80] 2019	Prospective Cross-sectional Australia E	Structured interview, nutritional assessment and medical record review	117 IBD		40% of patients in flare and 33% in remission avoided lactose.
Crooks et al. [66] 2019–2020	Single-center, prospective study UK E	Prospective questionnaire	208 patients with inactive UC	31% of patients reported a relationship between diet and UC onset.	21% avoided milk products.
Crooks et al. [83] 2019–2020	A prospective, cross-sectional, multi-center study UK E	29 questions questionnaire	91 UC 45 CD All ≥ 60 years		68% of patients followed dietary restrictions to prevent a flare of IBD; milk products were avoided in 23%.
Han et al. [53] 2020	A secondary analysis of the National Health Interview Survey 2015 US E	Survey	454 responders from a total population of 103,789 were ever told by health professionals that they have IBD	Consumption of milk in the past 30 days was similar among patients with IBD (71.29%; 95% CI 65.06–76.81) and without IBD (75.83%; 95% CI 75.10–76.55)	
Krela Kazmierczak et al. [67] 2020	Cross-sectional study Poland E	Questionnaire including consumption of milk and dairy products (qualitatively), especially before and after diagnosis of IBD.	208 adult IBD individuals (mean age 37.7 ± 14 years; 101 women) CD 103 UC 105		Milk consumption decreased after diagnosis (87% vs. 26.9%; *p* < 0.0001), but only in men. Percentage of people consuming dairy did not significantly decrease after diagnosis (100% vs. 90.4%; *p* = 0.0407).
Kamp et al. [84] 2020	Cross-sectional Study United States E	Dietary screening questionnaire, self-directed diet modifications, dietary beliefs questionnaire	147 adult IBD patients aged 18–35 with IBD 64% CD 90% females	69% of participants reported that diet modification could reduce IBD symptoms	66% of patients modified dairy consumption as a result of IBD.
Peters et al. [64], 2021	Case–control study The Netherlands D	FFQ by the nutritional department of Wageningen University	Groningen 1000IBD cohort and the Lifelines DEEP Cohort 493 IBD 207 UC 286 CD 61% females healthy population controls 1297		Compared to controls, patients with active disease consumed less cheese (27.2 ± 28.8 vs. 30.2 ± 26.8, *p* = 0.030), dairy (229 ± 185 vs. 285 ± 192, *p* < 0.001); UC patients ate less dairy (250 ± 171 vs. 285 ± 192, *p* = 0.014); CD patients consumed less cheese (26.2 ± 7.1 vs. 30.2 ± 26.8, *p* = 0.006) Patients in remission ate less dairy (227 ± 186 vs. 285 ± 192, *p* < 0.001).
Crooks et al. [66] 2021	Cross-sectional, multicenter study UK E	30-item questionnaire	255 British South Asians with IBD 154 UC 93 CD IBDU 8	42% of patients reported milk as a dietary trigger for IBD relapse.	49% of patients avoided milk products.
Sienkiewicz M. et al. [68] 2021	Cross-sectional Poland E	186-item FFQ	73 IBD 44% UC 103 healthy volunteers		IBD patients ate milk and dairy products less frequently (1.06 vs. 1.81, *p* < 0.001) than the control group.
Guida et al. [65] 2021	Prospective Italy E	Questionnaire on eating habits And food intolerances	167 IBD: 81 UC 86 CD 57.5% males the mean age 48.6 ± 16 years	Milk and dairy products were reported as symptom triggers by 57 patients (34.1%).	67.9% of participants avoided milk and dairy as they suspected, that they may be responsible for the occurrence of symptoms.
Vagianos et al. [79] 2022	Matched case–control longitudinal study Canada D	Biweekly online surveys for 1 year		61% of patients reported at baseline that food may induce flares, and 25% of those with active disease believed that food may have caused their current flare	Consumption of assessed foods in the previous 2 weeks between patients with active and inactive disease did not differ.
Cheng-Tzu Hsieh et al. 2022 [60]	Cross-sectional Taiwan E	Questionnaire developed by researchers from the UK, Japan, and Taiwan	50 UC 60% males 46.9 years	45.8% of patients reported, that diet triggered UC onset, and 48.0% of patients believed diet has ever caused flares.	59.2% of patients restricted consumption of milk and dairy.
Godala et al. [70] 2023	Prospective, questionnaire-based study Poland E	Questionnaire	82 IBD: 48 CD 34 UC 48.8% males 38.1 ± 11.6 years	47.8% believed, that milk and dairy should be avoided	28.9% of participants followed lactose-free diet, but 65.8% on lactose-free diet consumed cheese and yogurts.
Rana et al. [59] 2023	Cross-sectional study India E	Questionnaire	84 UC 38 years (24–49.3) 55.9% males		Amongst the predictors of flare consumption of dairy did not differ between patients and controls (88.2% and 75.8%).

* C: cohort study; D: case–controlled study; E: cross-sectional study; CCFA, Crohn’s Colitis Foundation of America; FFQ, food frequency questionnaire; IBD, inflammatory bowel disease; CD, Crohn’s disease; UC, ulcerative colitis; NR, not reported; GI, gastrointestinal; CD ^a^, CD without an ostomy or pouch; CD-O, CD with an ostomy; UC-P, UC with a pouch; ** authors used the Bonferroni method to correct for multiple comparisons, statistical significance was defined as *p* < 0.00039; items meeting this threshold are identified with an asterisk (**); items with a *p* value between 0.05 and 0.00039 are identified with #.

### 3.3. The Impact of Milk and Dairy Consumption on the IBD Course

Our search identified three studies providing data on the impact of milk and dairy products consumption on the IBD course measured with objective methods (Table 3).

Tasson et al. performed a cross-sectional study on 103 adult IBD individuals subdivided based on fecal calprotectin level into active disease and remission. The authors correlated the diet with disease activity assessed by fecal calprotectin. While they demonstrated that legumes and potatoes were inversely associated with disease relapse, and there was also a positive association between meat consumption and disease flare up, no relations were identified between flare and the consumption of dairy products [85]. Likewise, Vagianos et al., in the study described above in Section 3.2, found no difference in the intake of milk products between patients with active disease and remission. Although the majority of IBD patients believe that dietary items induce their disease relapses, this was not supported by the results of the study [79]. In turn, Komperød et al. [86] carried out a study including dietary intervention in a small group of CD patients in remission. Remission was defined as fecal calprotectin levels <250 mg/g. The most commonly reported dietary trigger of gastrointestinal symptoms in patients was cow’s milk. Patients followed a regimen of two weeks of a habitual diet period and two weeks of an elimination diet. The elimination diet was based upon two different approaches for patients with IBS: elimination of the most common food items reported as symptoms triggers, including milk, and the low-FODMAP (low-fermentable oligosaccharides, disaccharides, monosaccharides, and polyols) diet. The elimination diet was associated with a significant decline in symptom intensity. Despite the obvious limitations related to the short duration of intervention and the small number of patients, the study confirmed that milk is a common factor triggering the symptoms in CD patients. In a recently published abstract, dairy intake was not associated with inflammatory markers or symptomatic relapses. The mean weekly consumption of dairy products was similar between participants with a flare or no flare (30.9 oz vs. 35.4 oz; *p* = 0.70) [87].

Given the small number of studies and their limitations, primarily related to the size of the study groups and the length of follow-up, no firm conclusions can be drawn as to how dairy intake affects the course of IBD. It is also necessary to emphasize that assessing the impact on the course of the disease requires using objective activity methods. Currently, endoscopic examination remains the gold standard. Fecal calprotectin is considered a good non-invasive biomarker, although it is not free of limitations [88]. Hence, an evaluation of the dietary factor during the course of the disease would require, among other things, repeated endoscopic examinations, which, given their invasive nature, seem to be a barred. Nevertheless, the available studies do not suggest that dairy intake is associated with disease exacerbation.

**Table 3:** The impact of milk and dairy consumption on inflammatory bowel disease course.

Author Year	Study Design Country Quality Level *	Methods	Patients	Results
Tasson et al. [85] 2017	Cross-sectional Italy E	146-item FFQ on the intake of several foods over 1 year correlated with disease activity measured with fecal calprotectin	103 IBD 54 CD 49 UC 50 active 53 remission age 45.85 ± 14.2 years	No statistically significant associations were found between disease flare and the consumption of dairy.
Komperød et al. [86] 2018	Prospective Norway D	The intervention consisted of two weeks of a habitual diet and two weeks of an elimination diet.	16 CD in remission	Cow’s milk was the most commonly reported dietary trigger of GI symptoms (13 of 16 patients) The elimination diet was effective in CD patients in remission.
Vagianos et al. [79] 2022	Matched case–control longitudinal study Canada D	Biweekly online surveys for 1 year Self-reported flares	95 IBD flare 64 (67%) CD 31 (33%) UC 95 non-flare controls	Increased consumption of milk products in the past 2 weeks did not differ between flares (n = 25; 36%) and controls (n = 31; 33%, *p* = 0.257).

* D: case–controlled study; E: cross-sectional study; Crohn’s disease, CD; FFQ, food frequency questionnaire; GI, gastrointestinal; IBD, inflammatory bowel disease; ulcerative colitis, UC.

## 4. Summary

In our systematic review, we aimed to investigate the impact of milk and dairy consumption on several aspects of inflammatory bowel disease, from pathogenesis through its course to the patient’s beliefs and practices.

Dietary factors are repeatedly implicated in the pathogenesis of IBD and the increasing incidence of IBD is connected to global lifestyle changes. Further, the potential involvement of milk is debated based on experimental studies which have demonstrated that it may cause microbiome alterations and influence intestinal permeability [89]. After all, we did not find evidence that milk and dairy products can play a causative role in IBD pathogenesis. On the other hand, a recent large prospective study suggested that milk may have protective potential against the development of IBD.

Diet is taken into account as a factor influencing the course of the disease. For instance, in the Manitoba Living With IBD Study cohort, active inflammation measured by fecal calprotectin was less likely among individuals with a moderate Healthy Diet Score, whereas symptomatic IBD relapses were more likely [90]. Moreover, the consumption of fermented dairy products was associated with reduced serum CRP in healthy people [91]. Most patients (71%) presumed that their diet influenced their IBD [92]. One of the most controversial dietary agents, at least in the perception of patients with IBD, remains milk and dairy products, which is reflected by the studies on patients’ beliefs and practices. Milk and dairy products continue to be the most frequently excluded or restricted dietary items from IBD patients’ diets. In the majority of cases, it results from the self-reported relationship between milk and dairy product consumption and the onset of exacerbations of the disease symptoms. In turn, dietary restrictions may impact quality of life. A food-related quality of life [FR-QoL] questionnaire [93] systematically measures psychosocial factors surrounding eating and drinking, such as enjoying food, managing restrictions, and maintaining social relationships. FR-QoL is prevalent in IBD patients and, of particular significance, is associated with lower intakes of key nutrients of importance to IBD. For instance, patients with poorer FR-QoL had lower intakes of fiber (Q1 to Q5 difference = 2.1 g/d; 95% CI: 0.4–3.8; *p* = 0.048), calcium (192.6 mg/d; 95% CI: 112.5–272.6; *p* < 0.001), phosphorus (167 mg/d; 95% CI: 58–276; *p* = 0.041), and magnesium (34.4 mg/d; 95% CI: 9.3–59.4; *p* = 0.041) [94,95] when osteoporosis is present in 4–9% of both CD and UC patients [96].

Dietary restrictions were also associated with deficiencies in nutrients and their complications; yet, this was not the aim of our review. Nutrient deficiencies are generally prevalent in patients with IBD [97,98,99,100]. In the recent systematic review and meta-analysis, the intake of dairy, followed by the intake of legumes, fruit, and vegetables, was inadequate in inflammatory bowel disease patients [101]. Furthermore, an anti-inflammatory nutritional strategy, for instance, the Groningen anti-inflammatory diet (GrAID), consisting of lean meat, eggs, fish, fruit, vegetables, legumes, wheat, coffee, tea, and honey, also promotes the consumption of plain dairy, such as milk, yogurt, kefir and hard cheeses [102]. An awareness of the dietary restrictions of IBD patients is very significant, as patients assign more importance to diet in the treatment of their disease and less to medications compared to physicians [103].

The significance of diet in IBD does not end at this point, because diet may potentially impact the course of the disease and is a part of therapy. However, evidence on specific dietary factors triggering the IBD flare is missing. Moreover, the available guidelines do not recommend any particular diet for IBD patients. At the same time, milk and dairy products continue to be one of the basic food groups eaten currently, an important source of protein, micronutrients, and energy, but also if consumed by adults.

Dietary behaviors were similar among U.S. adults with and without IBD in the 2015 National Health Interview Survey (n = 33,626). For instance, dairy products were consumed once or more times per day by almost 65% of responders without IBD and 60% with IBD [104]. However, in the studies included in our review, milk and dairy consumption was often reduced by IBD patients.

**Figure 2:**
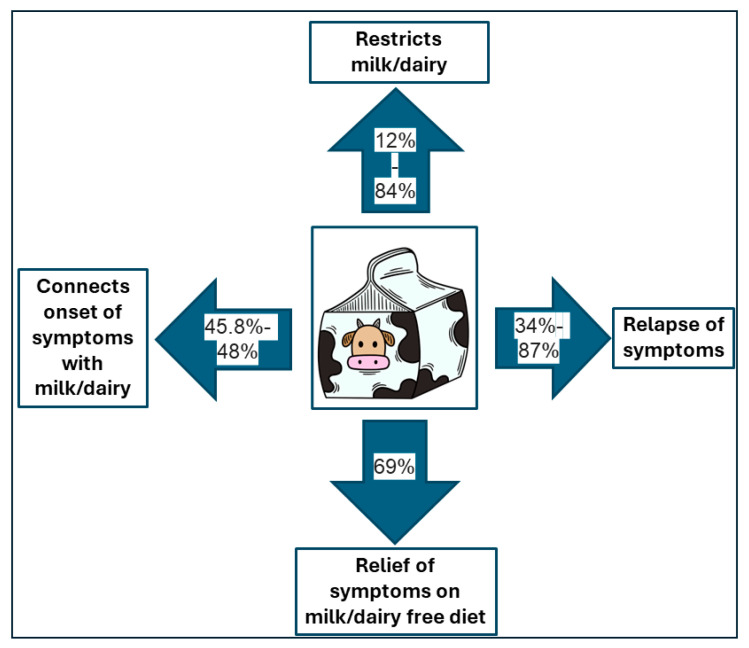
Interplay between milk/dairy consumption and inflammatory bowel disease.

Studies that assess the association between milk and dairy consumption and the onset and course of IBD are characterized by a great heterogeneity. Moreover, many studies are of low quality. Taking these aspects into account, it is impossible to draw firm conclusions. Nevertheless, based on this review, we can conclude that there is no clear evidence that milk and dairy consumption is associated with a higher risk of IBD. Studies on the effect of these dietary components on the course of the disease that have been carried out using objective markers of inflammation are few, and they do not confirm that milk and dairy consumption are associated with disease exacerbations. In contrast, there is a widespread perception among IBD patients that milk and dairy adversely affect the course of the disease. A large proportion of IBD patients stipulate a diet with restriction or exclusion of milk and dairy Figure 2.

The knowledge gap regarding the effect of milk and dairy on IBD in light of the beliefs and practices presented by patients points to the need for further, well-designed studies, the results of which will allow clear conclusions to be drawn. The use of objective markers of disease activity (fecal calprotectin, endoscopic activity) seems crucial, especially as patients with IBD may experience gastrointestinal symptoms even during endoscopic remission. In addition, the widespread use of dietary restrictions and elimination diets by patients with IBD who are at risk of malnutrition (which is not the focus of this article) highlights the importance of dietary education and counseling.

## 5. Limitations

Our systematic review has several limitations. The most important limitation of our work is the high heterogeneity of the research in terms of the design, population, and targets studied, as well as quality (see Table 1, Table 2 and Table 3). This makes it impossible to draw definitive conclusions. At the same time, given the importance of the issue, also in clinical practice, it points to the need for further research. Our literature search was limited to papers published after 1 January 2010. We aimed to review the most recent studies to reflect the current dietary beliefs and practices among IBD patients. Dairy food is a broad spectrum of milk-derived products that may have various properties, even if they are encountered in one group (like pure yogurt and yogurt with sweeteners and emulsifiers, cheese with different fat content, etc.). In the majority of studies, particular dairy products were not analyzed. We identified only two prospective, large cohort studies reporting data on milk and dairy products and IBD risk. The remaining studies were characterized by a small sample of IBD patients and their results have to be interpreted with caution. We identified only two studies that evaluated the impact of diet on IBD course and measured the activity of the disease with objective methods, including endoscopy, and fecal calprotectin. The remaining studies were usually based on self-reported symptoms. As the correlation between IBD clinical and endoscopic activity is poor, and there is a substantial group of patients with inactive IBD reporting gastrointestinal symptoms, this has to be taken into consideration. Further, the studies were conducted in different parts of the world, with various habitual consumptions of milk and dairy products. The majority of the studies were carried out in relatively small groups of patients. It has to be highlighted that multiple factors may influence diet, including factors related to IBD, namely disease phenotype, disease location, disease activity, disease duration, and also other factors, like age, gender, education, etc. Generally, they were not all considered. Additionally, the authors applied different types of questionnaires to assess dietary intake. Further, not all studies reported the cause of milk and dairy products exclusion or avoidance. In our review, we did not analyze lactose intolerance and cow milk allergies in IBD patients, despite the data suggesting that lactose intolerance is a risk factor for IBD.

## Data Availability

Not applicable.
